# Recommendations for the response against COVID-19 in migratory contexts under a closed border: The case of Colombia

**DOI:** 10.7705/biomedica.5512

**Published:** 2020-11-12

**Authors:** Julián Alfredo Fernández-Niño, Andrés Cubillos-Novella, Letza Bojórquez, Michael Rodríguez

**Affiliations:** 1 Departamento de Salud Pública, Universidad del Norte, Barranquilla, Colombia Universidad del Norte Departamento de Salud Pública Universidad del Norte Barranquilla Colombia; 2 Instituto de Salud Pública, Pontificia Universidad Javeriana, Bogotá, D.C., Colombia Pontificia Universidad Javeriana Instituto de Salud Pública Pontificia Universidad Javeriana BogotáD.C Colombia; 3 Departamento de Estudios de Población, El Colegio de la Frontera Norte, Tijuana, México Departamento de Estudios de Población El Colegio de la Frontera Norte Tijuana México; 4 Department of Family Medicine, The University of California, Los Ángeles, USA Department of Family Medicine The University of California Los Ángeles USA

**Keywords:** Emigration and immigration, public health, pandemics, coronavirus infections, COVID-19, Venezuela, Colombia, emigración e inmigración, salud pública, pandemias, infecciones por coronavirus, COVID-19, Venezuela, Colombia

## Abstract

Despite the positive response of Colombia's health system to the arrival of Venezuelan migrants, the new challenges that accompany the COVID-19 pandemic have triggered a closed-borders response that runs the risk of encouraging a negative view of migrants and increasing their health risks.

This manuscript discusses the recommendations that could be proposed in the case of a country with limited resources such as Colombia to respond to the needs of the Venezuelan mixed migrant flows.

## Resumen

A pesar de la respuesta positiva del sistema de salud de Colombia a la llegada de migrantes venezolanos, los nuevos desafíos que acompañan la pandemia de COVID-19 han desencadenado una respuesta de fronteras cerradas, con lo que se corre el riesgo de alentar una visión negativa de los migrantes e incrementar sus riesgos en salud. Este manuscrito discute las recomendaciones que podrían proponerse en el caso de un país con recursos limitados, como Colombia, para responder a las necesidades de una población vulnerable como la conformada por los flujos de migrantes mixtos venezolanos.

Since 2016, the number of people arriving in Colombia from Venezuela as part of mixed migration flows has increased sharply. The exodus of Venezuelans was the result of a combination of political, economic, and social circumstances that resulted in millions of them leaving the country for neighboring nations in precarious conditions and with few economic resources. This population movement, which began in 2015, is still going on: while in 2017 the Venezuelan population entering Colombia reached 796,234 people, in 2019 almost one and a half million (1,488,373) Venezuelans crossed the border into Colombia. Colombia currently hosts the largest number of Venezuelans living abroad: 1,771,237, of which 1,017,152 are irregular migrants ^1^.

Colombia and Venezuela share a long border of 2,219 km, as well as a similar cultural background; historically, this relationship has been close as reflected in the motto "fraternal countries" that is written on the bridges spanning the border.

The trade between the two countries is also very important. Some areas of the border are heavily populated and local people have traditionally moved back and forth between countries as part of their daily activities. Individuals and families move constantly in a pendular manner outnumbering those who cross the boundary to migrate or to seek asylum. The Wayuú indigenous group constantly moves in what is for them a transnational territory.

In the past years, cities in the Colombian side have become an important resource for Venezuelans who benefit from the chance to buy food and other goods that are difficult to find in their country contributing in turn to enhance the economic activity of the zone. According to the *Consejo Nacional de Política Económica y Social* (CONPES) ^2^, most people from Venezuela cross into Colombia to buy food (47%) and many do so to visit family members (12%) or as farmworkers (5%). As one side of the border depends economically and socially on the other, people who live close to it have kept on crossing despite the restrictions implemented at different times in the past.

Acknowledging the importance of addressing the public health issues associated with the massive arrival of members of mixed migration flows -i.e., population movements that combine refugees, asylum seekers, and economic migrants, and may also include unaccompanied minors, environmental migrants, smuggled persons, victims of trafficking, and stranded migrants, among others-, Colombia's *Ministerio de Salud y Protección Social* issued the *"Plan de respuesta sectorial al fenómeno migratorio"* ("Response plan of the health sector to address the migration phenomenon"). The Plan defined actions addressed to Colombian returnees from Venezuela, Venezuelans with both regular and irregular migration status, transborder commuters, and indigenous communities whose ways of life encompass both sides of the Colombia-Venezuela border ^3^. The Plan and subsequent regulations granted regular migrants access to healthcare *on par* with the Colombian population. Irregular migrants have a more limited access, but a strong policy of regularization starting in 2017 and kept since then facilitated healthcare access for those that were able to obtain a special permanence permit. All these strategies were political decisions based on the right to health and intended to protect the members of mixed migration flows.

Despite this positive response by the Colombian health system to the arrival of Venezuelan migrants, faced with the challenge of the COVID-19 pandemic Colombia decided to close the border with Venezuela on March 16, 2020. This decision might have inadvertently promoted a negative view of migrants and increased their health risks. Nationality should never be equated to illness and people coming from Venezuela, a country where only a few cases of the virus had been identified at the time of the border closure, were less likely to be carriers of COVID-19 than those arriving from Europe or some parts of the United States when the decision was made.

The attempt of sealing an international border has not been proved to be efficacious for transmission containment. Previous modelling of different strategies for outbreak mitigation shows that selectively targeting points of entry that are closer to the source of the outbreak might be effective while other strategies are less so ^4^. As Venezuela is currently not at the center of the COVID-19 outbreak, closing the border with that country is likely to have limited containment effects. Studies of influenza have shown that travel restrictions can delay, but not prevent, the expansion of the epidemic and containment needs to be based on a range of public health actions ^4^.

Given the scale of international traveling and everyday cross-border movement of people, it is very unlikely that a country will be able to remain completely isolated. Furthermore, these measures only make sense when a geographic area is completely free of the presence of the pathogen agent, which is probably not the case in a country such as Colombia with a permanent inflow of international travelers. Additionally, the border between Colombia and Venezuela is a notoriously porous one. Every day, people move across it through more or less hidden footpaths and the decision to close the official border crossings will encourage people to use those paths, which might require moving across jungle or through rivers increasing the risks of traveling.

Besides avoiding transmission, another reason for border closing might be to protect the local health services from potential overflow due to an increase in the number of people coming into the country. So far, however, there is no evidence of such an increase. A related reason could be the fear of the selective arrival of those who are already ill and cannot find healthcare in the deprived Venezuelan health system. In this regard, it is important to remember that while the increase in the number of Venezuelans in Colombia in the past few years has certainly tasked the healthcare system, according to an analysis by the World Bank the economic consequences could be offset in the medium and long term by the increase in productivity thanks to immigration ^5^. As for the second issue, there is no proof that the Venezuelan migration is mainly motivated by healthcare and even if this were the case in the context of the COVID-19 pandemic, it would be the time for the international community -not just for Colombia- to respond in accordance to the principle that a global public health concern requires a global response.

Additionally, in the last month, there has been an unexpected phenomenon of a flow of Venezuelan migrants returning to Venezuela whose number is not exactly known. These migrants returned forced by the economic difficulties derived from the obligatory social isolation and the subsequent loss of their employment. Venezuela's capacity to receive and protect them is also unknown.

What recommendations could then be proposed when a country with limited resources such as Colombia must respond to the needs of vulnerable populations like Venezuelan mixed migrant flows? We would like to advance four arguments:

First, the public health response should be centered on the two aims proposed by the World Health Organization of reducing human-to-human transmission and identifying, isolating, and caring for patients early.

Second, to achieve these aims in a mobile migrant population, allowing for their movement to take place in a "safe and orderly" context will make detection and control easier than when migrants are pushed towards irregular border crossings and forced to hide ^6^.

Third, as social distancing measures seem to be the best option for reducing transmission, it is imperative to provide migrants with the means to do so. If people are forced to wait at border areas without adequate protections, it will be impossible for them to self-quarantine when needed. On the other hand, providing adequate shelter and access to healthcare will make it easier to identify and treat cases and their contacts promptly. The ethical principle of "ought implies can" (i.e., that a person is not responsible for not adhering to law when she is not able to do so) means that for migrants to be able to self-quarantine or even hand-wash as recommended by the health authorities, they need to be provided with the means for it.

Fourth, restrictive measures in public health are only acceptable under the "harm principle", according to which individual freedom should only be limited when the common good is at stake. While every country has the right to define its migration policies and make decisions on who can enter through its borders, changing these decisions for public health reasons should only be done when the resulting strategy has been proven to be effective. Border closure, besides being a restrictive measure, runs the risk of encouraging racism and prejudice for which there should be zero tolerance.

In this sense, in the last month, the Colombian Government has been working in the inclusion of migrant population in the framework of the response against COVID-19 through a policy action plan that includes:

1. responsible and humanitarian political management of the border including the creation of humanitarian corridors for special populations such as students, indigenous people, pregnant women, and people with chronic diseases;
2. guarantee of access to healthcare services according to migratory status;
3. readjustment of international cooperation actions to respond to the current pandemic in migrant populations including cash money transfers, water, sanitation, and hygiene, care givers and care facilities adapted for isolation measures, food delivery, and hostels adaptation for homeless and walkers;
4. psychosocial attention for the most vulnerable population such as children and teenagers;
5. resources focalization in high impact municipalities such as Villa del Rosario (Norte de Santander), Maicao (La Guajira), and Soacha (Cundinamarca), and
6. information management and coordination for policy makers *(Ministerio de Salud y Protección Social. Balance general. Colombia incluye a los migrantes en su respuesta frente al COVID-19, pero necesita más apoyo internacional.* Bogotá; 2020).

This action plan follows some of the recommendations suggested by the World Health Organization (WHO), the United Nations High Commission for Refugees (UNHCR), and the International Organization for Migration (IOM).

Additionally, this plan complements the previously published "guidelines for the prevention, detection, and case management of COVID-19 for migrant population in Colombia" assigned to local and department governments, socio-healthcare teams, public social service centers, as well as healthcare providers involved in the care of migrants at risk, suspects or those with COVID-19 disease, especially for the detection, diagnosis, and isolation of suspected cases ^7^.

Besides, the Colombian government recognizes the importance of adopting inclusive actions and avoiding discrimination against the migrant population. The fact that there could be present barriers to healthcare, together with a discriminatory treatment, would lead to an environment where people diagnosed with the virus, infected or sick, may not receive the required treatment generating a very high level of virus spread ^7^.

However, the implementation of all these strategies continues to represent a great logistical and financial challenge for Colombia, especially since it is not known whether the flow of migrants will increase during the progress of the epidemic. Colombia has called for increased international aid and the consequences of the shortcomings of the Venezuelan health system on health and migration from that country are unpredictable.

Finally, measures that affect whole communities (in this case both Venezuelan migrants and Venezuelan and Colombian people living in the border regions) should be taken after discussion and coordination between national governments and policymakers in all sectors, as well as the public health community (academics, researchers, and practitioners). National governments must act in coordination and migrants should be included in all decisions that impact their health and wellbeing ^8^. A deeper binational dialogue and effective cooperation between Colombia and Venezuela are therefore imperative. Regional and international agencies and States should rebalance migration policymaking to give greater importance to health including public health experts and decision-makers in high-level forums for migration policymaking. Leaders and health professionals should be fully involved in conversations about the macroeconomic forces affecting population mobility and participate in multi-sectorial budgeting and planning for migrant programs.

To conclude, we hope that the COVID-19 pandemic will not become an excuse for the implementation of restrictive migration policies and that no additional burden will be placed on already vulnerable populations. If anything, this should be the opportunity to develop strategies to ensure that the migrant population, regardless of age, gender, or legal status, receives equitable healthcare in a framework of truly universal coverage ^8^.
